# Non-endoscopic Mechanical Endonasal Dacryocystorhinostomy

**Published:** 2011-07

**Authors:** Mohammad Etezad Razavi, Morteza Noorollahian, Alireza Eslampoor

**Affiliations:** 1 Khatam-al-Anbia Eye Research Center, Mashhad University of Medical Sciences, Mashhad, Iran; 2 Emam Reza Hospital, Mashhad University of Medical Sciences, Mashhad, Iran

**Keywords:** Endonasal Dacryocystorhinostomy, Mechanical, Nasolacrimal Duct Obstruction

## Abstract

To circumvent the disadvantages of endoscopic dacryocystorhinostomy such as small rhinostomy size, high failure rate and expensive equipment, we hereby introduce a modified technique of non-endoscopic mechanical endonasal dacryocystorhinostomy (NE-MEDCR). Surgery is performed under general anesthesia with local decongestion of the nasal mucosa. A 20-gauge vitrectomy light probe is introduced through the upper canaliculus until it touches the bony medial wall of the lacrimal sac. While directly viewing the transilluminated target area, a nasal speculum with a fiber optic light carrier is inserted. An incision is made vertically or in a curvilinear fashion on the nasal mucosa in the lacrimal sac down to the bone using a Freer periosteum elevator. Approximately 1 to 1.5 cm of nasal mucosa is removed with Blakesley forceps. Using a lacrimal punch, the thick bone of the frontal process of the maxilla is removed and the inferior half of the sac is uncovered. The lacrimal sac is tented into the surgical site with the light probe and its medial wall is incised using a 3.2 mm keratome and then excised using the Blakesley forceps. The procedure is completed by silicone intubation. The NE-MEDCR technique does not require expensive instrumentation and is feasible in any standard ophthalmic surgical setting.

## INTRODUCTION

The standard surgical procedure for treatment of nasolacrimal duct obstruction (NLDO) is dacryocystorhinostomy (DCR) which restores normal lacrimal outflow. This procedure can be performed via an external or endonasal approach. The external approach is the gold standard for acquired NLDO and has largely remained unchanged.^1^ Success rates for this procedure are often reported to be over 90% at many subspecialty units.^2^,^3^ However, the cutaneous incision and disruption of the medial canthal ligaments with resultant lacrimal pump dysfunction have been reported as significant disadvantages.^4^,^5^

Endonasal DCR was first proposed by Caldwell^6^ in 1893, who used an electrical burr to create a middle meatal osteotomy in the area marked by a metal probe. Advantages of endonasal DCR over the external approach include less morbidity, reduced intraoperative bleeding, shorter operative time and preservation of lacrimal pump function since the orbicularis oculi, presac fibers and the medial canthal tendon are not disrupted.^7^ Furthermore, the endonasal approach avoids an external scar and provides the possibility of simultaneous management of nasal and sinus abnormalities through the same surgical approach. Disadvantages of endonasal DCR are small rhinostomy size, higher failure rates, more expensive equipments and a steep learning curve.^8^,^9^

Herein, we introduce a modified technique of mechanical endonasal DCR which does not require specialized and expensive equipments including an endoscope.

## SURGICAL TECHNIQUE

**Figure 1:**
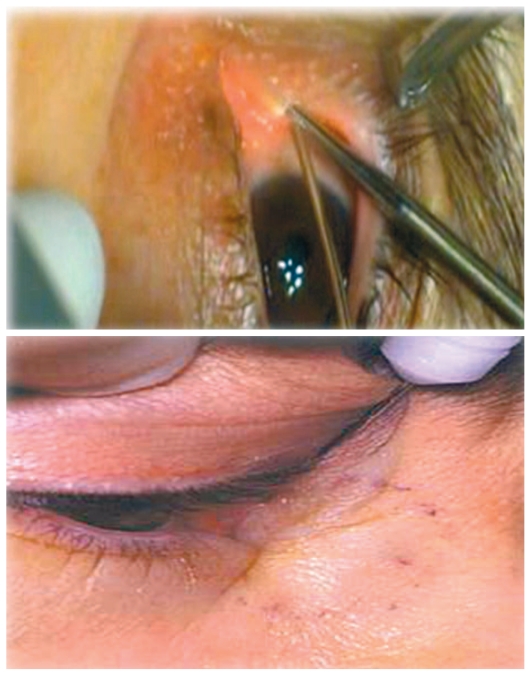
Insertion of the vitrectomy light probe.

**Figure 2:**
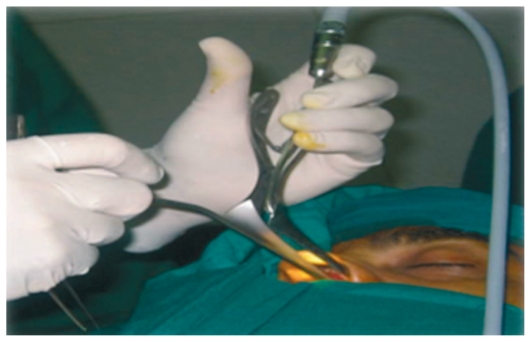
Direct view of the nasal cavity using a lighted nasal speculum.

The procedure is usually performed under general anesthesia. First the nasal cavity is decongested using 6 cotton pledgets soaked in nasal phenylephrine 0.25% for 5 minutes. A 20-gauge vitrectomy light probe is introduced through the upper canaliculus until it touches the bony medial wall of the lacrimal sac and is then turned downward (Fig. 1). The right-handed surgeon takes position on the right side of the patient for both right and left sided endonasal DCR and directly views the transilluminated target area (Fig. 2) through a nasal speculum with 7.5 cm long blades and a fiber optic light carrier (Storz endoscope instruments, Karl Storz, Germany).

**Figure 3:**
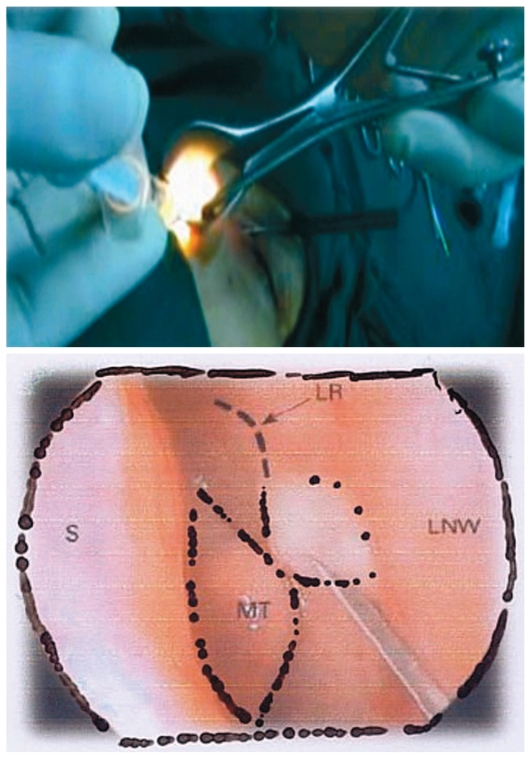
The site of adrenaline injection MT, middle turbinate; LR, lacrimal ridge; S, nasal septum; LNW, lateral nasal wall

**Figure 4:**
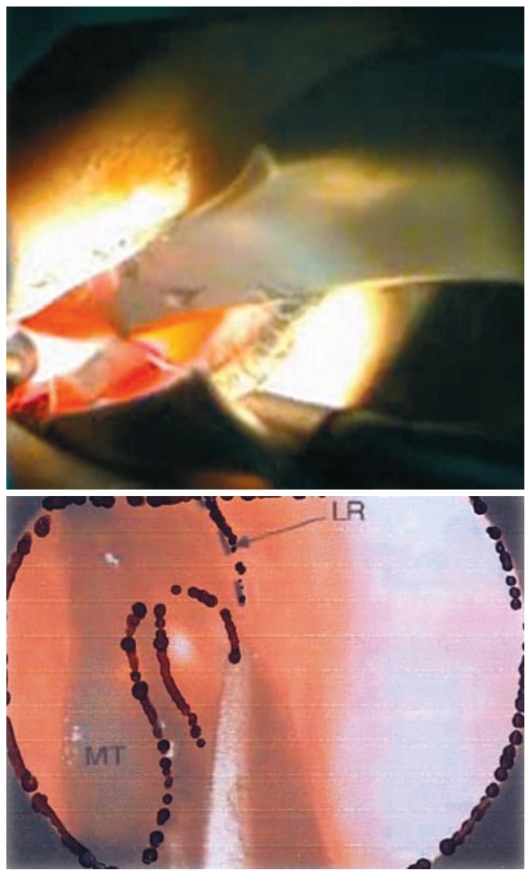
A Freer elevator is used for making an incision on the nasal mucoperiosteum at the lacrimal ridge. MT, middle turbinate; LR, lacrimal ridge.

A 1 cm^2^ area on the lateral nasal wall just anterior to the middle turbinate is infiltrated with 2% lidocaine plus epinephrine 1:100,000 until bleaching is evident (Fig. 3). A Freer periosteum elevator is used to incise the nasal mucosa by using the light probe in the lacrimal sac as a guide. The incision is made vertically or in a curvilinear fashion down to the bone (Fig. 4).

**Figure 5:**
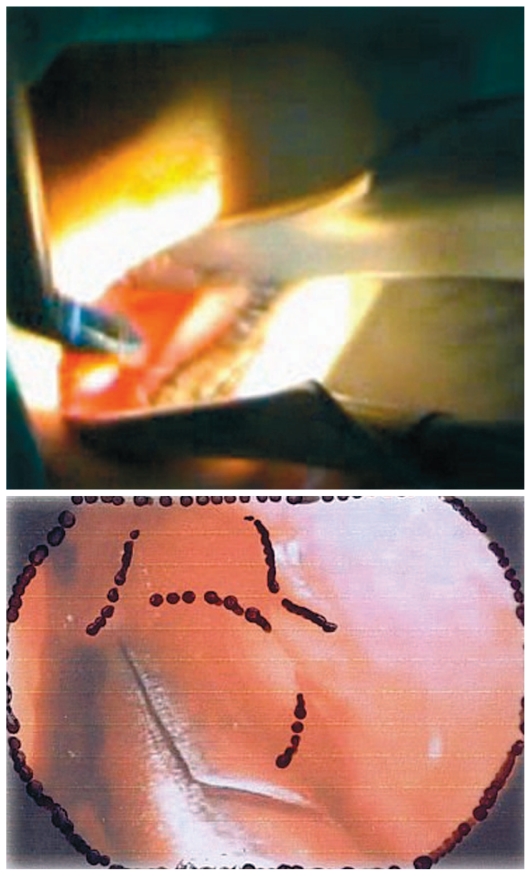
Straight Blakesley forceps grasping the flap.

**Figure 6:**
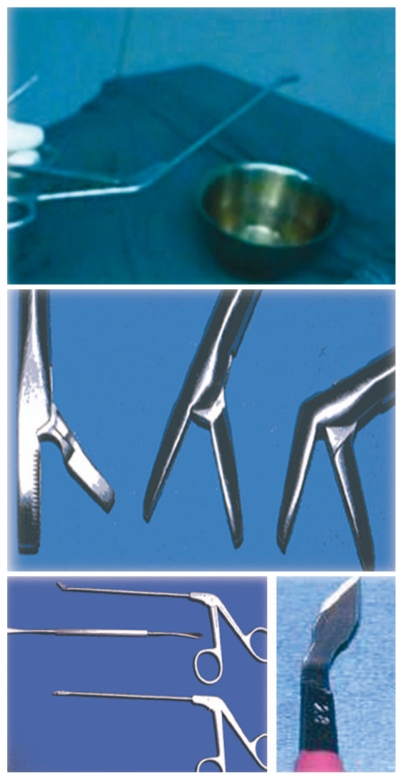
Straight and up-biting Blakesley forceps and freer elevator (top, center, and bottom left), and keratome (bottom right).

**Figure 7:**
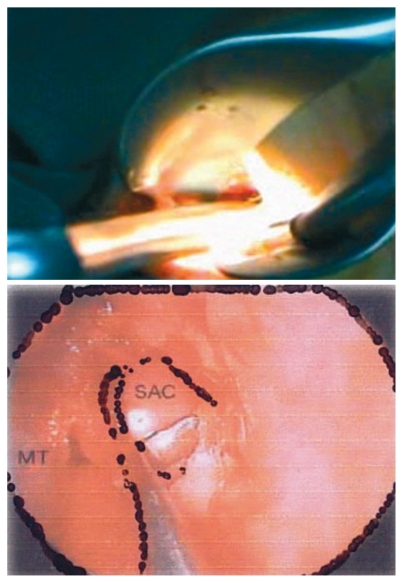
The Kerrison rongeur taking large bites of the thick maxillary bone. MT, middle turbinate; SAC, lacrimal sac

**Figure 8:**
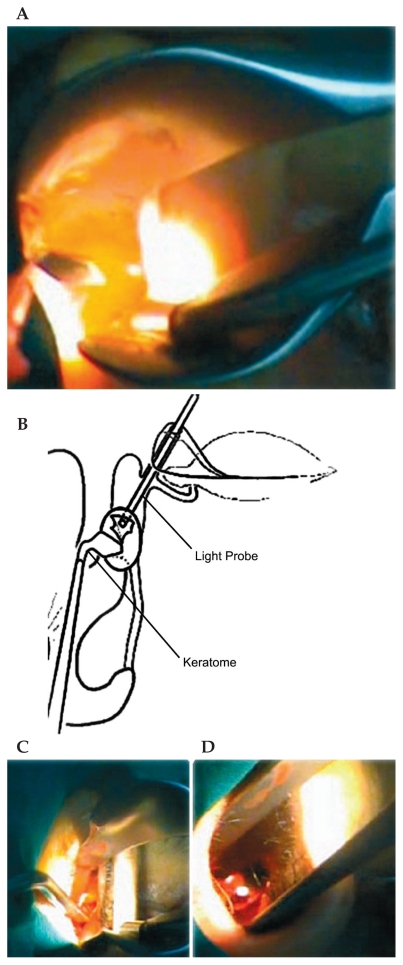
Lacrimal sac incision (A & B) and lacrimal sac flap removal (C & D).

**Figure 9:**
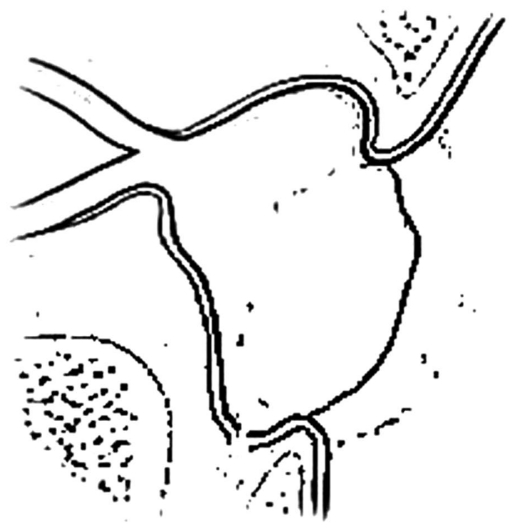
Rhinostomy on the lateral nasal wall.

Approximately 1 to 1.5 cm of nasal mucosa is removed using Blakesley or Takahashi forceps (Storz endoscope instruments, Karl Storz, Germany). Once the lacrimal fossa is exposed, the thin lacrimal bone is elevated from the posterior half of the lower lacrimal sac up to the insertion of the uncinate process (Figures 5 and 6). Using a forward-biting lacrimal punch, the hard thick bone of the frontal process of the maxilla is then removed and the inferior half of the sac is uncovered (Fig. 7). Once the lacrimal sac mucosa is exposed, the lacrimal sac is tented into the surgical site using the light probe followed by incision of the medial wall of the lacrimal sac using a 3.2 mm keratome and than excision with a Blakesley forceps (Figures 8 and 9).

**Figure 10:**
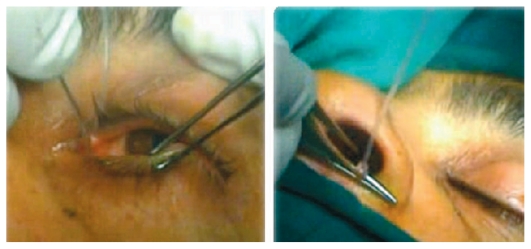
Silicone intubation.

Finally bicanalicular silicone tubes are introduced into both canaliculi and retrieved from the nasal cavity using a hemostat. Metal ends of the tubes are cut and the tube is tied with a square knot and retained in the nasal cavity (Fig. 10).

## DISCUSSION

The technique presented herein avoids complications associated with lasers and drills such as thermal damage which can cause scarring and thus predispose to DCR failure.^10^–^12^ The other disadvantage of ablative laser-assisted surgery is that a lacrimal sac mucosal biopsy cannot be taken.

Endoscopic removal of the lacrimal bone and the thick frontal process of the maxilla, which together form the anterior lacrimal crest, can be technically difficult. Previously it was believed that the lacrimal sac is anterior to or below the axilla of the middle turbinate with little extension above it, but it is now known that the lacrimal sac lies mainly above the level of the axilla.^13^ Therefore, removal of the thick bone along the anterior edge of the lacrimal sac is important to achieve unobstructed lacrimal drainage.

Bone removal by laser is tedious and has been associated with a high rate of surgical failure. Concomitant use of a drill and a rongeur has been advocated to obtain a larger rhinostomy and prevent closure.^14^ With our technique, the use of a Hajek-Koeffler forward-biting punch achieved fast and practical removal of bone with no need for sophisticated and expensive instruments. Compared with drilling, this procedure is atraumatic, very simple and controllable.

Primary failure rates of external DCR have been less than 10%.^15^,^16^ Primary failure rates of endoscopic DCR range from 10 to 33%.^3^ The failure rate of NE-MEDCR, based on our experience, is similar external DCR with an overall success rate of 96% making this procedure a suitable alternative to external DCR with less dependency on complex instrumentation.
